# Inhibition of Triggering Receptor Expressed on Myeloid Cell-1 Alleviates Acute Gouty Inflammation

**DOI:** 10.1155/2019/5647074

**Published:** 2019-12-06

**Authors:** Yonglong He, Qibin Yang, Xiuxiu Wang, Aimin Jia, Wenguang Xie, Jingguo Zhou

**Affiliations:** ^1^Hospital of Chengdu University of Traditional Chinese Medicine, Chengdu 610075, China; ^2^Department of Rheumatology and Immunology, The Affiliated Hospital of North Sichuan Medical College, Nanchong 637000, China; ^3^Department of Hematology, The Affiliated Hospital of North Sichuan Medical College, Nanchong 637000, China; ^4^Department of Rheumatology and Immunology, The First Affiliated Hospital of Chengdu Medical College, Chengdu 610500, China

## Abstract

Gout is a prevalent form of aseptic inflammation caused by the deposition of monosodium urate (MSU) crystals in joints or tissues. Triggering receptor expressed on myeloid cell-1 (TREM-1) is a superimmunoglobulin receptor expressed on innate immune cells including granulocytes, monocytes, and macrophages. TREM-1 serves as a link between innate immunity and adaptive immunity, playing a crucial role in regulating inflammation and immune response. The purpose of this study was to investigate the potential role of TREM-1 in THP-1 cells and peripheral blood mononuclear cells (PBMCs) from patients with gouty arthritis (GA). In the current study, we found that the mRNA and protein levels of TREM-1 increased in PBMCs from GA patients and soluble TREM-1 in plasma as well. In addition, an increased level of TREM-1 was observed in THP-1 treated with monosodium urate (MSU) in vitro, along with upregulation of proinflammatory cytokines. Moreover, upon specific inhibition of TREM-1, Toll-like receptor 4 (TLR-4), and myeloid differentiation factor 88 (MyD88), the levels of MyD88 and proinflammatory cytokines were decreased after MSU challenge in THP-1 cells. Interestingly, inhibition of TLR-4 could enhance the effect of TREM-1 inhibitor in MSU-induced inflammation. Taken together, our findings suggested that TREM-1 could accelerate MSU-induced acute inflammation. Inhibition of TREM-1 may provide a new strategy for alleviating acute gouty inflammation.

## 1. Introduction

Gouty arthritis (GA) is aseptic inflammatory arthritis characterized by the deposition of monosodium urate (MSU) crystals in joints and tissues. Gout often has the unique feature of the recurrent acute attacks and spontaneous remission and is involved in various kinds of immunocytes including monocytes and macrophages [1]. A previous study reported that gout was associated not only with metabolism and inflammation but also with immunity, especially the innate immune signaling pathway [2]. Currently, Toll-like receptors (TLRs) and Nod-like receptor protein 3 (NLRP3) inflammasome signaling pathways are widely related to MSU-induced inflammation [3, 4]. TLR-4 is the most thoroughly investigated receptor in the TLR family [5]. MyD88 and nuclear factor- (NF-) *κ*B are the downstream effectors of the TLR-4 signaling pathway [6]. Our previous data showed that the TLR-4/NF-*κ*B/IL-1*β* signaling pathway played a crucial role in the pathogenesis of acute inflammation in primary gout patients [7].

Triggering receptor expressed on myeloid cell-1 (TREM-1), which is a superimmunoglobulin receptor expressed on innate immune cells including granulocytes, monocytes, and macrophages, plays a crucial role in innate and adaptive immunity and acts to initiate inflammation or to amplify inflammatory responses [8, 9]. The previous study showed that TREM-1 is significantly related to inflammation [10]. Another marvelous feature of the TREM-1 was the release of soluble TREM-1 [11]. Increasing evidences have verified that the levels of TREM-1 and sTREM-1 were remarkably increased in sepsis [12] and autoimmune diseases, including rheumatoid arthritis [13], systemic lupus erythematosus [14], and primary antiphospholipid syndrome [15]. Therefore, TREM-1 may be an important mediator of inflammation. Several studies showed that TREM-1 was increased in gout patients and animal models [16–18]. Studies have shown that TREM-1 modulates the signaling pathways of pattern recognition receptors (PRRs), including Toll-like receptors (TLRs) and Nod-like receptors (NLRs) [19, 20]. However, whether the function of TREM-1 was involved in gouty inflammation via TLR-4 signaling pathway was not clarified.

In this study, we found that the levels of TREM-1 and sTREM-1 were increased in patients with gouty arthritis. In addition, we confirmed that TREM-1 enhanced the function of TLR-4 in MSU-induced inflammatory response in vitro. Therefore, these findings suggest that TREM-1 could contribute to the development of MSU-induced acute inflammation. Blockade of TREM-1 might have an effective strategy in the treatment of GA.

## 2. Materials and Methods

### 2.1. Patients

One hundred and twenty-six male patients with primary GA who visited the Department of Rheumatology of the Affiliated Hospital of North Sichuan Medical College from January 2018 to May 2019 were enrolled. Sixty-six cases of acute gouty arthritis (AGA) patients were diagnosed according to the classification criteria of the American College of Rheumatology (ACR) [21]. Sixty cases of intercritical gouty arthritis (IGA) were diagnosed with complete remission of AGA and a normal C-reactive protein (CRP) or erythrocyte sedimentation rate (ESR). Seventy-two healthy age-matched males without hyperuricemia were enrolled as healthy control (HC). These participants had no history of infection, other autoimmune diseases, hematopathy, cancer, or nephropathy. The laboratory and clinical characteristics of the patients are shown in Table 1. The Ethics Committee of the Affiliated Hospital of North Sichuan Medical College approved the research protocol, and all patients filled up informed consent forms to participate in the study. The research was performed in accordance with the principles of the current version of the Declaration of Helsinki.

### 2.2. PBMCs and Plasma Isolation

All samples were obtained from venous blood including AGA, IGA, and HC. The plasma was separated by centrifugation, and the peripheral blood mononuclear cells (PBMCs) (2 × 10^6^ cells) were isolated using density gradient centrifugation. The PBMCs and plasma were saved at −80°C for following gene or protein measurement.

### 2.3. Cell Culture

Human THP-1 cells were incubated in 1640 medium supplemented with 10% FBS (UTAH, USA) at 37°C, in a 95% humidity incubator. The THP-1 cells were induced with PMA (100 ng/mL, Louis, USA) in 10 cm petri dishes. The cells were cultured in 6 well plates and incubated with black control (equal amount of culture medium) and MSU (100 *μ*g/mL, Sigma, USA) with or without the TREM-1 inhibitor (Shanghai, China), TLR-4 inhibitor (InvivoGen, USA), and MyD88 inhibitor (MedChem, USA) for 6 hours; then cell and supernatant were harvested for gene or protein measurement.

### 2.4. Real-Time Quantitative PCR

Total RNA were extracted from PBMCs and THP-1 cells using RNAiso plus reagent and were transcripted to the complementary DNA by reverse transcription kits (TaKaRa, Japan). Real-time quantitative PCR (RT-qPCR) reactions were completed with the ABI QuantStudio 12K Flex (Applied Biosystems, USA) with SYBR Premix using specific primers: 40 cycles of 95°C for 15 s and 60°C for 60s. The value of each sample was detected two times and was averaged. The primer sequences were synthesized by a biological engineering company (Shanghai, China). The specific sequences of the primers were as follows: 5′-TTG TCT CAG AAC TCC GAG CTG C-3′ (forward) and 5′-GAG ACA TCG GCA GTT GAC TTG G-3′ (reverse) for TREM-1; 5′-GAC CTG TCC CTG AAC CCT A-3′ (forward) and 5′-CTC CCA GAA CCA AAC GAT G-3′ (reverse) for TLR-4; 5′-GTG GGG ACT ACG ACC TGA AT-3′ (forward) and 5′-GGG GCA CGA TTG TCA AAG AT-3′ (reverse) for NF-*κ*B p65; 5′-ATA TGC CTG AGC GTT TCG AT-3′ (forward) and 5′-GCG GTC AGA CAC ACA CAA CT-3′ (reverse) for MyD88; and 5′-CTC CCA GAA CCA AAC GAT G-3′ (forward) and 5′-GAG CTA CGA GCT GCC TGA CG-3′ (reverse) for *β*-actin. The expression of target gene was normalized using house-keeping gene *β*-actin and quantified the gene levels by the 2^−*ΔΔ*Ct^ methods [22]. Each experiment was repeated three times independently.

### 2.5. Western Blot

Total proteins from PBMCs and THP-1 cells (5 × 10^5^ cells) were extracted using minute™ Total Protein Extraction Kit (cat#SD001, invent, China) containing phosphatase or protease inhibitors. The protein concentrations were measured by BCA assay kit (Beyotime, China). Proteins (100 *μ*g) were separated by 10% sodium dodecyl sulfate-polyacrylamide gel electrophoresis and transferred to the polyvinylidene fluoride (PVDF) membrane (Bio-Rad). The PVDF membrane was blocked in 5% nonfat milk (Beyotime, China) for one hour at room temperature; then the membranes were incubated with the anti-TLR-4 (1 : 500, Abcam, USA, cat#ab13867), anti-TREM-1 (1 : 100, Santa Cruz, USA, cat#sc-2293450), anti-MyD88 (1 : 1000, Cell Signaling Technology (CST), USA, cat#4283), and *β*-actin (1 : 1000, CST, USA, cat#5125) overnight at 4°C and rinsed for ten minutes with Tris-Buffered Saline tween (TBST) buffer and then incubated with secondary antibody conjugated to horseradish peroxidase (1 : 3000, CST, USA, cat#7074) for 1 h at room temperature. Proteins were detected utilizing enhanced chemiluminescence Western blot substrate (Engreen, Beijing, China). The image was analyzed and quantified with Image-Pro Plus 6 software (Media Cybernetics Company).

### 2.6. Enzyme-Linked Immunosorbent Assay

Plasma and cell supernatant were centrifuged and stored at −80°C. IL-6 (cat#D6050), IL-8 (cat#D8000C), TNF-*α* (cat#DTA00D), IL-1*β* (cat#DLB50), and MCP-1 (cat#DCP00) levels in supernatant exposed to MSU crystals with or without different inhibitors and the plasma level of TREM-1 (cat#DTRM10C) were analyzed and determined using enzyme-linked immunosorbent assay (ELISA) kits from R&D Systems (USA) in accordance with the manufacturer's instructions. The optical density was measured using a microplate reader (Model 3550, Bio-Rad). A standard curve for IL-1*β*, IL-6, TNF-*α*, IL-8, and MCP-1 was established using a known concentration of IL-1*β*, IL-6, TNF-*α*, IL-8, and MCP-1 by plotting the optical density relative to the log of the concentration.

### 2.7. Statistical Analysis

The SPSS 16.0 software and GraphPad Prism 5.0 software were used to analyze the data. The data were presented as the means ± SEM. Numerical variables between the two groups were tested using an unpaired *t*-test. Multiple comparisons were performed using one-way analysis of variance (ANOVA) in combination with the Bonferroni posttest. Differences were considered significant at *p* < 0.05.

## 3. Results

### 3.1. TREM-1 and Soluble TREM-1 Were Increased in Patients with Gouty Arthritis

**Figure 1:**
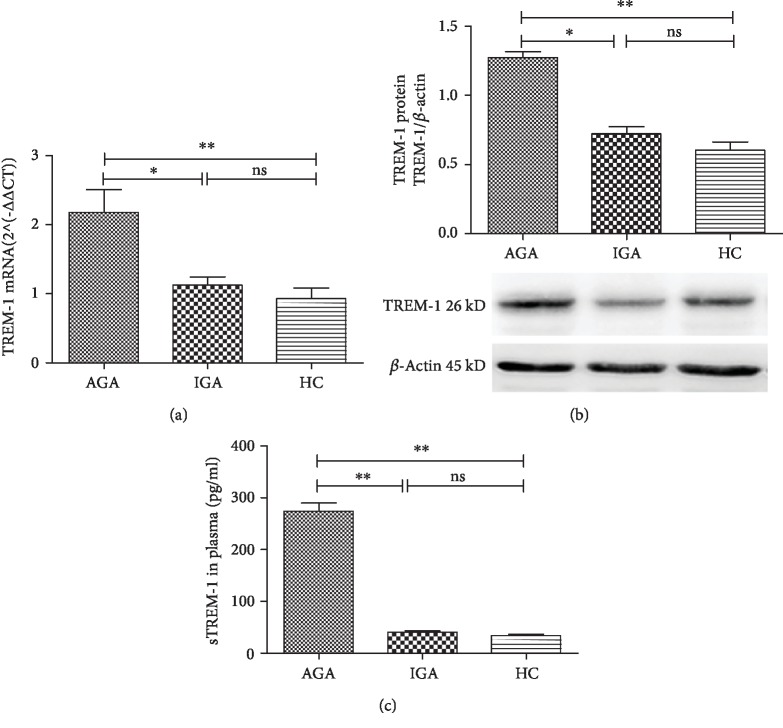
TREM-1 participated in the development of GA. (a) The level of TREM-1 mRNA in PBMCs of patients with AGA (*n* = 66), IGA (*n* = 60), and HC (*n* = 72) was detected by RT-qPCR. (b) The level of TREM-1 protein in PBMCs of patients with AGA, IGA, and HC was measured by Western blot (6 cases each group). (c) The level of sTREM-1 in plasma from patients with AGA (*n* = 32), IGA (*n* = 27), and HC (*n* = 27) was detected by ELISA. Data were presented as the mean ± SEM. ^∗^*p* < 0.05; ^∗∗^*p* < 0.01. ns: no significance.

We detected whether TREM-1 and sTREM-1 were altered in patients with AGA and IGA. We found that the TREM-1 mRNA level was significantly upregulated in the AGA group compared with the IGA or HC group (Figure 1(a)). Although the level of TREM-1 mRNA from IGA group had an increased change compared with those from the HC group, the change had no statistical significance (Figure 1(a)). The TREM-1 protein in the AGA group was significantly increased than that in the IGA group or HC group (Figure 1(b)). Furthermore, the TREM-1 protein of the IGA group was comparable with that of the HC group (Figure 1(b)). Plasma soluble TREM-1 level was remarkably increased in AGA group compared with the IGA group or HC group, whereas there was no difference between IGA group and HC group (Figure 1(c)). Our results indicated that TREM-1 and sTREM-1 may play an important pathological role in gouty arthritis.

### 3.2. The Level of TREM-1 Was Upregulated by MSU in THP-1 Cells

**Figure 2:**
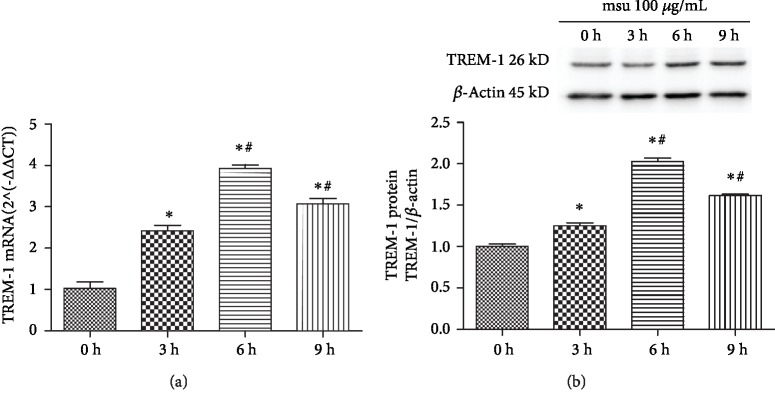
The level of TREM-1 was upregulated with MSU in THP-1 cells. The THP-1 cells were stimulated with MSU for 0, 3, 6, and 9 hours. (a) The level of TREM-1 mRNA was detected by RT-qPCR. (b) The level of TREM-1 protein was detected by Western blot. Data were presented as the means ± SEM of three wells per group. Individual experiments were conducted three times, and the representative data were shown. ^∗^*p* < 0.05, compared to the blank control; ^#^*p* < 0.05, compared to the group treated for three hours.

To further verify the role of TREM-1 in MSU-induced inflammation, we measured the TREM-1 level at different time points post MSU stimulation in THP-1 cells. We found that the mRNA (Figure 2(a)) and protein (Figure 2(b)) levels of TREM-1 increased at 3 h and reached a peak at 6 h. Our results suggested that TREM-1 was upregulated in MSU-induced inflammation.

### 3.3. Specific TREM-1 Inhibitor (LP17) Inhibited MSU-Induced Proinflammatory Cytokine Release in THP-1 Cells

**Figure 3:**
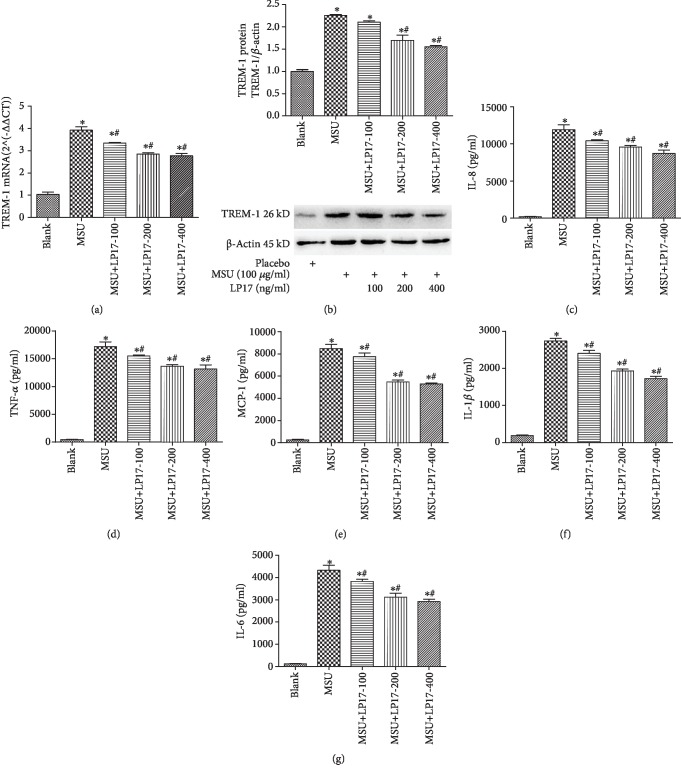
Specific TREM-1 inhibitor (LP17) inhibited MSU-induced proinflammatory cytokine release in THP-1 cells. (a) The level of TREM-1 mRNA was detected by RT-qPCR. (b) The level of TREM-1 protein was detected using Western blot. (c–f) Protein levels of IL-1*β*, TNF-*α*, MCP-1, IL-6, and IL-8 were measured by ELISA. Data were presented as the means ± SEM of three wells per group. Individual experiments were conducted three times. ^∗^*p* < 0.05, compared to the blank group; ^#^*p* < 0.05, compared to the MSU group.

To investigate the effect of TREM-1 to inflammatory cytokine production from the THP-1 cells upon MSU stimulation. We detected the TREM-1 and proinflammatory cytokine level in THP-1 cells, which were treated with different concentration (0, 100, 200, or 400 ng/mL) of a specific TREM-1 inhibitor (LP17) before MSU stimulation. We found that the mRNA (Figure 3(a)) and protein (Figure 3(b)) levels of TREM-1 were significantly suppressed in a dose-dependent manner. In addition, we found that the levels of IL-8, TNF-*α*, MCP-1, IL-1*β*, and IL-6 were inhibited in a dose-dependent manner (Figures 3(c)–3(g)). Our data suggested that the inhibition of TREM-1 by LP17 suppressed MSU-induced proinflammatory cytokine release in a dose-dependent manner.

### 3.4. Interaction between TREM-1 and TLR-4 in MSU-Induced Inflammation

**Figure 4:**
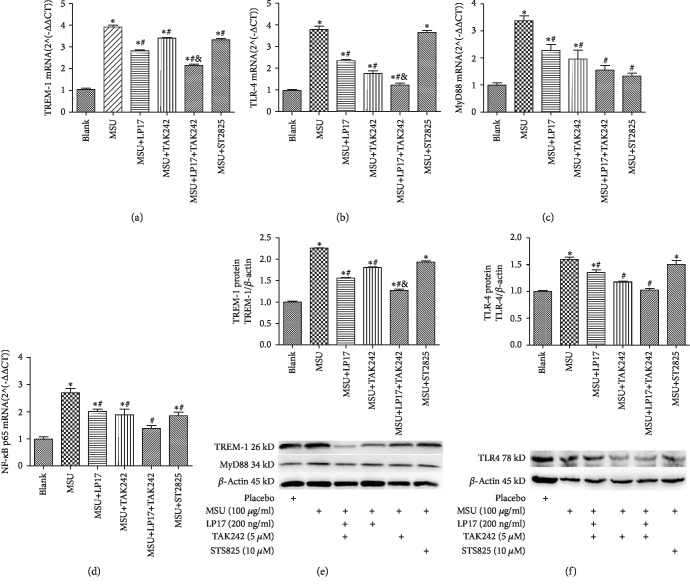
Interaction effect of TREM-1 and TLR-4. (a–d) The mRNA levels of TREM-1, TLR-4, MyD88, and NF-*κ*B p65 were analyzed by RT-qPCR in THP-1 cells. (e, f) The TREM-1, MyD88, and TLR-4 protein levels were measured using Western blot. Data were presented as the means ± SEM of three wells per group. Individual experiments were conducted three times. ^∗^*p* < 0.05 compared with the blank group; ^#^*p* < 0.05 compared with the MSU group; ^&^*p* < 0.05 compared with the MSU+LP17 group and the MSU+TAK242 group.

Previous studies demonstrated that TREM-1 on monocytes/macrophages could amplify the inflammatory effects in infectious diseases, and interaction between TREM-1 and TLR-4 could enhance the TLR-4 signaling pathway activity leading to multiple proinflammatory mediator secretion [8]. Based on the study above, we investigated whether the TREM-1 and TLR-4 signaling pathways have a synergistic effect in THP-1 cells treated with or without TREM-1 inhibitors, TLR-4 inhibitors, and MyD88 inhibitors for 2 h before MSU stimulation and whether deficiency of TREM-1 or TLR-4 could downregulate the secretion of proinflammatory cytokines including IL-1*β*, TNF-*α*, IL-6, IL-8, and MCP-1. In the in vitro experiment, the levels of TREM-1, TLR-4, MyD88, and NF-*κ*B p65 were detected. As shown in Figure 4, compared with the blank control, the levels of TREM-1 (Figure 4(a) and 4(e)), TLR-4 (Figure 4(b) and 4(f)), MyD88 (Figure 4(c)), and NF-*κ*B p65 (Figure 4(d)) were significantly upregulated after MSU treatment. However, LP17, as the specific TREM-1 inhibitor, could decrease mRNA and protein levels of TREM-1 in THP-1 cells treated with MSU. Of note, LP17 could downregulate the levels of TLR-4, MyD88, and NF-*κ*B p65. Moreover, in addition to TLR-4, we found that the TLR-4 inhibitor (TAK22) significantly inhibited the upregulation of TREM-1, MyD88, and NF-*κ*B p65 in MSU-induced inflammation. Besides, ST2825, as the specific inhibitor of MyD88 which was the key adapter protein of the TLR-4 signaling pathway, reduced the levels of TREM-1, MyD88, and NF-*κ*B p65 in response to MSU in THP-1 cells compared with the group of MSU stimulation alone.

### 3.5. Synergism of TREM-1 and TLR-4 in the Proinflammatory Cytokine Production

**Figure 5:**
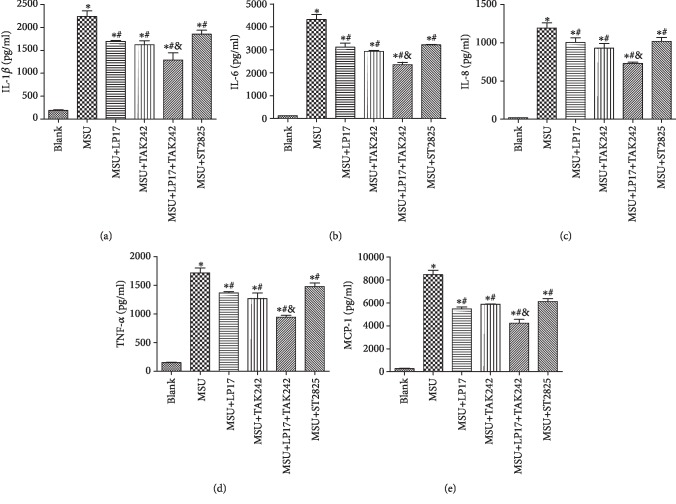
TREM-1 synergizes with TLR-4 in proinflammatory cytokine production. (a–e) Protein levels of IL-1*β*, IL-6, IL-8, TNF-*α*, and MCP-1 were tested by ELISA. Data were presented as the means ± SEM of three wells per group. Individual experiments were conducted three times. ^∗^*p* < 0.05 compared with the blank group; ^#^*p* < 0.05 compared with the MSU group; ^&^*p* < 0.05 compared with the MSU+LP17 group and the MSU+TAK242 group.

The levels of proinflammatory cytokines (IL-1*β*, IL-6, IL-8, TNF-*α*, and MCP-1) in cultural supernatants were detected by ELSIA post MSU with a specific inhibitor, including TREM-1, TLR-4, and MyD88. Compared with the blank group, MSU dramatically promoted upregulation of IL-1*β* (Figure 5(a)), IL-6 (Figure 5(b)), IL-8 (Figure 5(c)), TNF-*α* (Figure 5(d)), and MCP-1 (Figure 5(e)) levels. However, upregulation of those proinflammatory cytokines could be decreased by LP17 or TAK242. Furtherly, no significant difference was observed between the MSU+LP17 and MSU+TAK242 groups. Interestingly, we found that the lowest levels of cytokines were observed in the combination of the LP17 and TAK242 groups. Together, these results suggested that the TLR-4 inhibitor could improve MSU-induced inflammatory response, which could be synergized by the TREM-1 inhibitor.

## 4. Discussion

The innate immune system initiated inflammatory responses through the recognition of MSU crystals as a danger signal [23]. TREM-1 was a transmembrane protein receptor of a new immunoglobulin superfamily and was mainly expressed on the surface of macrophages and neutrophils [8]. The previous evidence indicated that MSU could induce macrophages and leukocytes from the murine peritoneal cavity, increasing TREM-1 expression on these cells and secretion of proinflammatory cytokines such as IL-1*β* and MCP-1 [16]. Our results demonstrated significantly elevated levels in THP-1 cells with MSU treatment in vitro. Our results were consistent with the increased TREM-1 level in synovial fluid mononuclear cells from acute gouty arthritis after MSU stimulation [18]. Moreover, sTREM-1 levels were remarkably increased in the AGA patients compared with IGA patients and healthy controls. Previous studies have shown that upregulation of monocyte membrane TREM-1 during endotoxemia was closely related with an increased release of sTREM-1 in human [24, 25]. It also occurred in various immune diseases, including rheumatoid arthritis [13, 26] and systemic lupus erythematosus [15]. Therefore, those data suggested that plasma sTREM-1 might serve as a reliable biomarker for acute gouty arthritis and autoimmune diseases as well.

The proinflammatory cytokines played a crucial role in the pathological process of gout [27]. In our in vitro experiment, the levels of IL-1*β*, IL-6, IL-8, TNF-*α*, and MCP-1 were significantly increased in THP-1 cells after MSU challenge. Several experiments have demonstrated that inhibition of TREM-1 reduced inflammation [28, 29]. LP17, as the specific inhibitor of TREM-1, is a greatly conserved sequence present in humans and mice [18]. The regulation of the TREM-1 signaling pathway by LP17 could significantly affect the progression of inflammation and immune diseases [24]. In the present study, we found that TREM-1 was significantly inhibited by LP17 in a dose-dependent manner. Furthermore, we found that inhibition of TREM-1 by LP17 could reduce the secretion of proinflammatory cytokines in MSU-induced inflammation. It was consistent with a previous study that triptolide inhibited the inflammatory response in rheumatoid arthritis by modulating the TREM-1 signaling pathway [29]. Taking our findings together, it indicated that TREM-1 enhanced inflammatory response and blockade of TREM-1 might be a potential treatment for GA.

Numerous researches have demonstrated that TREM-1 was upregulated by innate signaling that activated TLRs [8, 30]. TLR-4 was a cell-surface receptor which could also recognize “danger signals” such as MSU crystals [31]. Blockade of TREM-1 with a recombinant chimeric protein could prevent an increase in LPS-induced inflammatory cytokine production in THP-1 cells [32]. Furthermore, the inflammatory response could be eliminated after blockade of TREM-1 and TLR-4 [32]. Besides, silencing the macrophage TREM-1 resulted in the alteration of key receptors and effector proteins of the TLR-4 signaling pathway [33]. These results suggested that TREM-1 and TLR-4 may have a synergistic effect in the inflammatory response. However, a synergistic effect between TREM-1 and TLR-4 in MSU-induced inflammatory response has not been clarified. Previous research reported that the expression of MyD88, IL-1*β*, and MCP-1 was significantly decreased in the TREM-1 knockdown RAW264.7 cells, whereas the expression of TLR-4 was not changed [33]. In the current study, TREM-1 inhibitors could give rise to downregulation of TLR-4, MyD88, and NF-*κ*B p65. The reasons may be resulted from the recognition of different ligands or cell type. Moreover, we observed that the upregulation of TREM-1 was inhibited in response to MSU in THP-1 cells with the TLR-4 inhibitor (TAK22) or MyD88 inhibitor (ST2825) compared with MSU stimulation alone. Our results were similar to previous reports that TLR-4 activation also leads to upregulation of TREM-1 expression in a MyD88-dependent manner [8, 25]. Additionally, the levels of proinflammatory cytokines in the supernatant, including IL-1*β*, IL-6, IL-8, TNF-*α*, and MCP-1, were significantly downregulated in the specific inhibitor group. Our results were consistent with those findings that TREM-1 was involved in the secretion of proinflammatory cytokines including TNF-*α*, IL-*β*, and IL-8 [8]. Interestingly, the levels of TREM-1 and proinflammatory cytokines were significantly reduced in the combination of TREM-1 and TLR-4 inhibitors compared with those specific inhibitors alone. It has been shown that TREM-1 modulated the TLR-4 pathway by regulating MyD88 expression and NF-*κ*B activation [33]. In addition, NF-*κ*B activation was required for TREM-1 expression [34]. These results agreed with reports that simultaneous activation of TREM-1 and TLR-4 led to synergistic production of proinflammatory mediators [35, 36].

There are several limitations in this study. First, the level of TREM-1 and its related key molecules was not observed in PBMCs from gouty arthritis at different stages after the combination of specific inhibitors with MSU treatment. Second, we should verify the difference between the decreased expressions of TLR-4 after TREM-1 inhibitor treatment and unaltered TLR4 in the TREM-1-deficient RAW264.7 cell. Third, the biological function of sTREM-1 in gouty inflammation was still unclear. Though sTREM-1 served as a novel biomarker, more experiments are necessary to measure its function in gouty inflammation.

In conclusion, our data indicated that TREM-1 was highly expressed in THP-1 cells and in PBMCs from acute gouty arthritis and plasma soluble TREM-1 was viewed as a potential biomarker for acute gout. Downregulation of TREM-1 could contribute to alleviating inflammation, but more researches need to be investigated. It would be a novel strategy of prevention and therapy to patients with gouty arthritis.

## Figures and Tables

**Table 1:** Clinical and laboratory characteristics of the subjects.

Items	AGA (*n* = 66)	IGA (*n* = 60)	HC (*n* = 72)	*F* value	*p* value
Age (years)	42.65 ± 11.50	38.24 ± 8.36	38.62 ± 10.22	0.74	0.478
Disease duration (years)	8.6 ± 4.38	8.36 ± 4.03	NA	NA	NA
BMI (kg/m^2^)	25.79 ± 3.32	24.68 ± 3.08	23.36 ± 4.47	7.17	<0.001
SBP (mmHg)	128.63 ± 14.94	124.52 ± 12.36	124.98 ± 11.23	1.94	0.07
DBP (mmHg)	85.72 ± 11.13	80.36 ± 10.58	78.45 ± 8.89	2.88	0.18
Tophi, *n* (%)	13 (19.70%)	NA	NA	NA	NA
Renal calculus, *n* (%)	10 (15.15%)	7 (11.67%)	NA	NA	NA
Diabetes mellitus, *n* (%)	5 (7.58%)	3 (5.00%)	NA	NA-	NA
ESR (mm/h)	14.40 ± 16.22	3.67 ± 6.28	3.30 ± 6.12	21.98	<0.001
WBC (×10^9^/L)	9.51 ± 3.09	7.02 ± 1.85	8.82 ± 5.59	6.61	<0.001
Granulocyte (×10^9^/L)	6.90 ± 2.93	4.46 ± 1.49	6.46 ± 3.43	34.13	<0.001
Lymphocyte (×10^9^/L)	1.89 ± 0.56	1.94 ± 0.81	2.95 ± 1.73	17.69	<0.001
Monocyte (×10^9^/L)	0.56 ± 0.21	0.42 ± 0.17	0.71 ± 0.35	39.72	<0.001
TG (mmol/L)	2.50 ± 1.20	2.40 ± 1.80	1.30 ± 0.50	19.71	<0.001
TC (mmol/L)	4.59 ± 1.49	4.92 ± 0.81	4.42 ± 0.52	3.99	0.194
HDL (mmol/L)	1.10 ± 0.40	1.20 ± 0.40	1.40 ± 0.50	8.39	<0.001
LDL (mmol/L)	2.40 ± 0.90	2.80 ± 0.80	2.30 ± 0.70	6.92	0.0012
VLDL (mmol/L)	1.20 ± 0.60	1.24 ± 0.64	0.70 ± 0.60	16.53	<0.001
Apo B100 (mmol/L)	0.91 ± 0.25	0.96 ± 0.22	0.74 ± 0.14	21.12	<0.001
Glucose (mmol/L)	6.02 ± 1.31	5.94 ± 1.1	4.92 ± 0.51	20.52	<0.001
Uric acid (*μ*mol/L)	518.38 ± 123.12	479.41 ± 123.84	329.13 ± 50.98	69.85	<0.001
CRP (mg/L)	31.37 ± 72.39	1.87 ± 3.23	1.54 ± 2.55	11.09	<0.001

BMI: body mass index; SBP: systolic blood pressure; DBP: diastolic blood pressure; ESR: erythrocyte sedimentation rate; WBC: white blood cell; TG: triglyceride; TC: total cholesterol; HDL: high-density lipoprotein cholesterol; LDL: low-density lipoprotein cholesterol; VLDL: very low-density lipoprotein cholesterol; Apo A1: Apolipoprotein A1; Apo B100: Apolipoprotein B100; CRP: C-reactive protein.

## Data Availability

The data used to support the findings of this study are available from the corresponding author upon request.
